# Immunomodulatory mechanisms of abatacept: A therapeutic strategy for COVID-19

**DOI:** 10.3389/fmed.2022.951115

**Published:** 2022-07-25

**Authors:** Dinglong Yang, Hetong Li, Yujing Chen, Weiping Ren, Mingjie Dong, Chunjiang Li, Qiang Jiao

**Affiliations:** ^1^Second Clinical Medical College, Shanxi Medical University, Taiyuan, China; ^2^School of Public Health, Xi'an Jiaotong University, Xi'an, China; ^3^Department of Orthopedics, The Second Hospital of Shanxi Medical University, Taiyuan, China

**Keywords:** abatacept, COVID-19, target genes, immune and inflammatory responses, cytokine storm

## Abstract

Coronavirus disease 2019 (COVID-19) caused by coronavirus-2 (SARS-CoV-2) infection has rapidly spread throughout the world and become a major threat to human beings. Cytokine storm is a major cause of death in severe patients. Abatacept can suppress cytokines used as antirheumatic drugs in clinical applications. This study analyzed the molecular mechanisms of abatacept treatment for COVID-19. Differentially expressed genes (DEGs) were identified by analyzing expression profiling of abatacept treatment for rheumatoid arthritis (RA) patients and SARS-CoV-2 infection patients. We found that 59 DEGs were upregulated in COVID-19 patients and downregulated following abatacept treatment. Gene set enrichment analysis (GSEA) and Gene Ontology (GO) analysis showed that immune and inflammatory responses were potential regulatory mechanisms. Moreover, we verified 8 targeting genes and identified 15 potential drug candidates for the treatment of COVID-19. Our study illustrated that abatacept could be a promising property for preventing severe COVID-19, and we predicted alternative potential drugs for the treatment of SARS-CoV-2 infection.

## Introduction

Coronavirus disease 2019 (COVID-19) is an infectious and global pandemic disease caused by coronavirus-2 (SARS-CoV-2) (1). The clinical symptoms were characteristically severe acute respiratory syndrome and hyperinflammatory immune response (2). T-cell immune responses are at the forefront of eliciting potent antiviral responses (3). Uncontrolled viral infection in advanced diseases resulting from insufficient T-cell responses may lead to systemic inflammation and severe lung damage (4, 5). Clinical manifestation is represented by an immune defense-based protective phase first and is characterized by broad inflammation subsequently, which may lead to multiple organ failure (MOF) in severe patients (6). The drastic cytokine storm in severe patients is one of the causes leading to death (7). The widespread use of biological therapies in rheumatoid arthritis has shown a rapid resolution. There was evidence that the combination of anti-cytokine agents and systemic corticosteroid therapy had lower mortality rates (8). While there are no effective drugs for COVID-19 presently, the current treatment option for severe patients is symptomatic supportive therapy.

Many rheumatoid drugs have shown potential for the treatment of COVID-19 based on their pharmacological properties. Abatacept, a T-cell selective co-stimulation modulator specifically binding to CD80 and CD86, was approved for use in rheumatoid arthritis (RA) with good efficacy in clinical application. Abatacept modulates T-cell activation and prevents the production of cytokines and downstream immune responses in RA (9–11). Therefore, neutralizing inflammatory factors in cytokine release syndrome (CRS) based on the pathophysiology of rheumatic diseases will be of great value in preventing disease progression in severe COVID-19 patients (12). Steroid hormone is widely used in COVID-19 treatment, but its side effects are also obvious, such as femoral head necrosis. Drug repurposing is a strategy for identifying new indications for approval. Evidence has been provided for abatacept as a candidate therapeutic approach to prevent severe COVID-19 (13). However, the mechanism of abatacept acting on COVID-19 remains unclear. Meanwhile, foreknowledge of the similar drug-target network providing alternative treatment strategies for COVID-19 patients is still challenging.

In this study, we conducted a bioinformatics analysis to explore the role of abatacept in the treatment of COVID-19 and provide more alternative anti-COVID-19 therapeutic drugs of similar pharmacological effects in the absence of sophisticated drugs for COVID-19. We also performed gene set enrichment analysis (GSEA), Gene Ontology (GO) analysis, and miRNA-mRNA network construction to reveal potential molecular mechanisms of abatacept preventing excessive inflammation and MOF. We also identified 8 abatacept targeting genes in the COVID-19 treatment. Finally, we identified the top 15 drug candidates combined with 8 targeting genes, offering a therapeutic strategy for severe COVID-19.

## Materials and methods

### Data acquisition

The RNA-seq data (GSE151161, GSE152418, and GSE157103) were downloaded from the Gene Expression Omnibus (GEO) (https://www.ncbi.nlm.nih.gov/geo/) database. The GSE151161 dataset consists of 76 whole blood samples from 38 RA patients before and post abatacept treatment. The GSE152418 dataset included 34 peripheral blood mononuclear cell (PBMC) samples from 17 COVID-19 patients and 17 normal controls, which were used for analysis. The GSE157103 dataset involved 126 whole blood leukocytes from 100 COVID-19 patients and 26 normal controls, which were used for validation (Table 1).

**Table 1:** Information for selected microarray datasets.

**GEO accession**	**Platform**	**Samples**				**Source tissue**	**Attribute**
		**RA with abatacept week 0**	**RA with abatacept week 12**	**COVID-19**	**Normal controls**		
GSE151161	GPL24676	38	38	–	–	Whole blood	Test set
GSE152418	GPL24676	–	–	17	17	Whole blood (PBMC)	Test set
GSE157103	GPL24676	–	–	100	26	Whole blood (Leukocytes)	Validation set

### Identification of differentially expressed genes

We used R (version 3.6.3) package “DESeq2” (version 1.28.1) to determine differentially expressed genes (DEGs) (14). Threshold values were considered as follows: Padj < 0.05, | log2FoldChange (logFC)| > 1. A Venn diagram was used to identify common DEGs between GSE151161 and GSE152418. The ggplot2 R package (version 3.3.3) was used to graph the Venn diagram and Volcano plot.

### Gene set enrichment analysis

Gene set enrichment analysis, GSEA (http://software.broadinstitute.org/gsea/msigdb/index.jsp), was performed using the R package clusterProfiler (3.14.3) for identifying potential hallmarks of abatacept treatment whole blood and reactomes of SARS-CoV-2-infected PBMC (15). The adjusted *P*-value (< 0.05), FDR q value (< 0.25), and normalized enrichment score (|NES| > 1) were used to classify enrichment differences of function in each phenotype.

### Enrichment analysis of DEGs

Gene Ontology analysis of DEGs (biological processes, cellular components, and molecular functions) was performed using the R software (version 3.6.3) and the R package clusterProfiler (version 3.14.3). R package org.Hs.eg.db (version 3.10.0) was used for ID conversion. GO for the immune system process was analyzed using the CluGO (version 2.5.7) (16) apps of Cytoscape Software (version 3.8) (17). Padj < 0.05 was considered as the threshold. The top 15 GO terms with the smallest Padj value were presented.

### Identification of hub genes

The protein–protein interaction (PPI) network of DEGs was established using the STRING (https://cn.string-db.org/) online tool (score > 0.15) (18). The Cytoscape software was used to visualize the PPI network and identify hub genes (top 14). The GeneMANIA database (http://genemania.org/) was used to predict potential genes interacting with hub genes and analyze their functions (19).

### Differential protein and mRNA expression of hub genes in multiple tissues

We further explore the RNA and protein expression of 8 hub genes in multiple tissues in the Human Protein Atlas (HPA) database (https://www.proteinatlas.org/) (20). The RNA expression data were in transcripts per kilobase of exon model per million mapped reads (TPM) format from the Consensus dataset.

### Single-cell mRNA expression of hub genes in lung

In the HPA database, we explored the single-cell RNA expression of 8 hub genes in lung tissues. The cell types included macrophages, alveolar cells type 2, T cells, granulocytes, fibroblasts, club cells, respiratory ciliated cells, endothelial cells, and alveolar cells type 1.

### Differential mRNA expression of hub genes in multiple immune cells

In the HPA database, the RNA expression of 8 hub genes in 19 immune cell subtypes was explored. The RNA expression data were in TPM format from the HPA dataset. The 19 types of immune cells consisted of plasmacytoid dendritic cell (DC), myeloid DC, memory CD8 T cell, natural killer (NK) cell, total PBMC, basophil, eosinophil, neutrophil, classical monocyte, non-classical monocyte, intermediate monocyte, regulatory T cell, gd T cell, mucosal-associated invariant T (MAIT) cell, memory CD4 T cell, naïve CD4 T cell, naive CD8 T cell, memory B cell, and naive B cell.

### Prediction of the miRNA-mRNA interaction

The NetworkAnalyst database (https://www.networkanalyst.ca/) was used to predict target miRNAs of hub genes (21). In addition, the R ggalluvial package (version 0.12.3) was used to construct the mRNA-miRNA co-expressed interaction networks based on the interaction information.

### Relationship between hub gene and immunocyte-related gene expression

The Spearman's rank correlation coefficient was used to analyze the relationship between the expression of hub genes and immunocyte-related genes, including CD3, CD4, CD8, CD19, CD20, CD27, CD28, CD56, CD11b, CD66b, CCL4, and CCL5. R (version 3.6.3) was used for analysis and the R ggplot2 package (version 3.3.3) was used for visualization. Threshold values were considered as follows: ^*^p < 0.05 indicates a mild correlation and ^**^p < 0.01 indicates a moderate correlation.

### Identifying drug candidates

We used the Drug Signatures database DSigDB tool of Enrichr (https://maayanlab.cloud/Enrichr/) to identify drug candidates targeting hub genes for COVID-19 treatment (22). The top 15 drug candidates were selected according to combined scores, from highest to lowest.

### Statistical analysis

All statistical analysis was carried out using R (version 3.6.2). The Spearman's rank correlation coefficient was used for correlation analysis. A Wilcoxon rank-sum test was used to compare the expression of hub genes in two groups. The hypothetical test was two-sided in all tests, and a *p*-value of <0.05 was considered statistically significant.

## Results

### Identification of DEGs after SARS-CoV-2 infection and abatacept treatment

**Figure 1:**
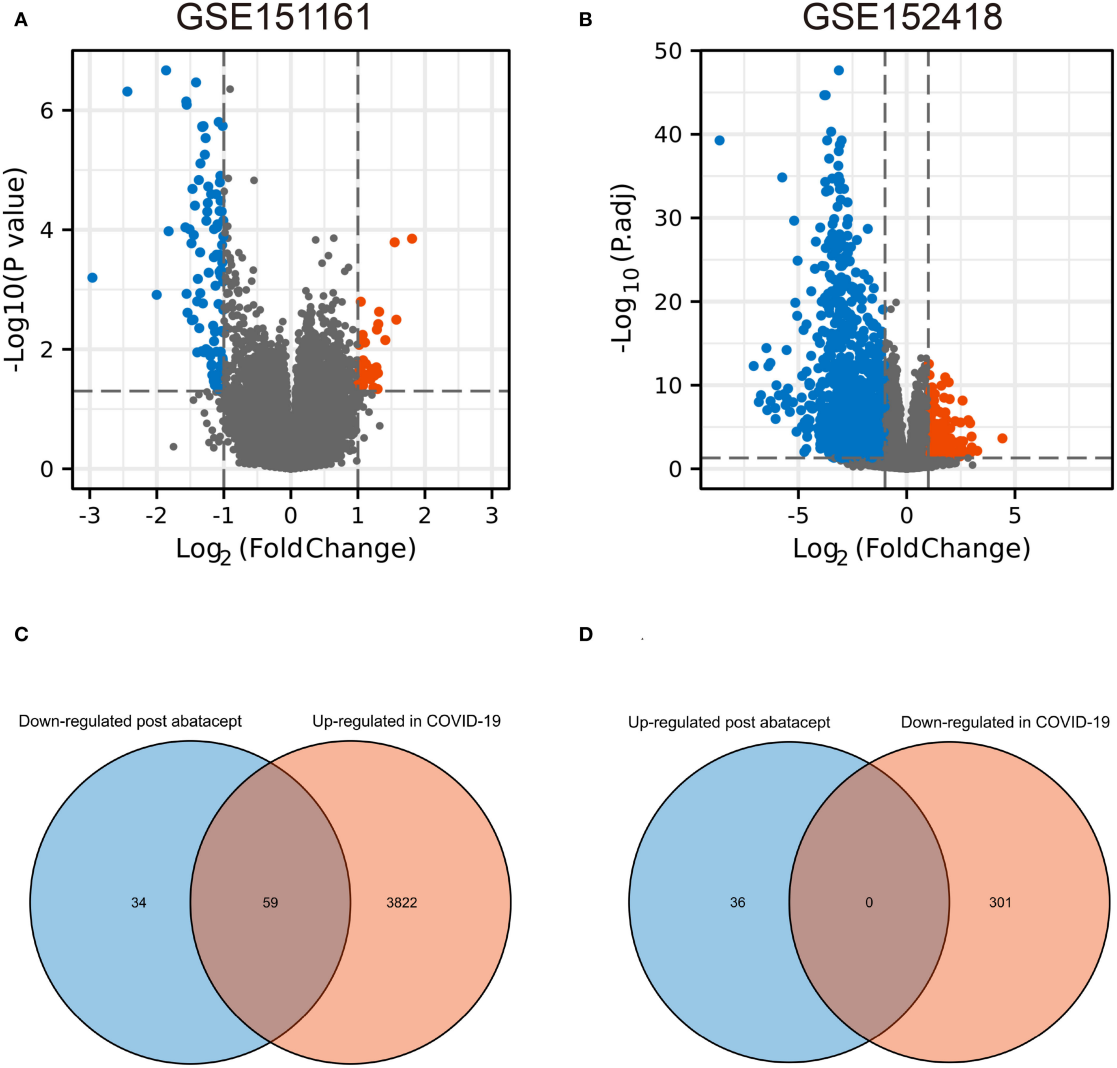
The volcano map and Venn diagram between two datasets. **(A)** Differential expression genes (DEGs) of GSE151161. Ninety-three genes were downregulated and 36 genes were upregulated post abatacept treatment (12 weeks) in rheumatoid arthritis patients. **(B)** Differential expression genes of GSE151161. Three hundred and one genes were down-regulated and 3,881 genes were upregulated in COVID-19 patients. **(C)** Fifty-nine DEGs were upregulated in COVID-19 patients and downregulated post abatacept treatment. **(D)** Zero DEG was downregulated in COVID-19 patients and upregulated post abatacept treatment.

The volcano map showed 36 upregulated genes and 93 downregulated genes in GSE151161 after the abatacept treatment for 12 weeks in patients with RA (Figure 1A). COVID-19 caused significant gene expression changes in the samples of PBMC. A total of 4,182 DEGs were identified from GSE151161, including 3,881 upregulated genes and 301 downregulated genes (Figure 1B). The Venn plot showed that 59 DEGs were both upregulated in COVID-19 patients and downregulated post abatacept treatment (Figure 1C). Conversely, there were no DEGs downregulated in COVID-19 patients and upregulated post abatacept treatment (Figure 1D).

### GSEA showed the immune-inflammatory response activity

**Figure 2:**
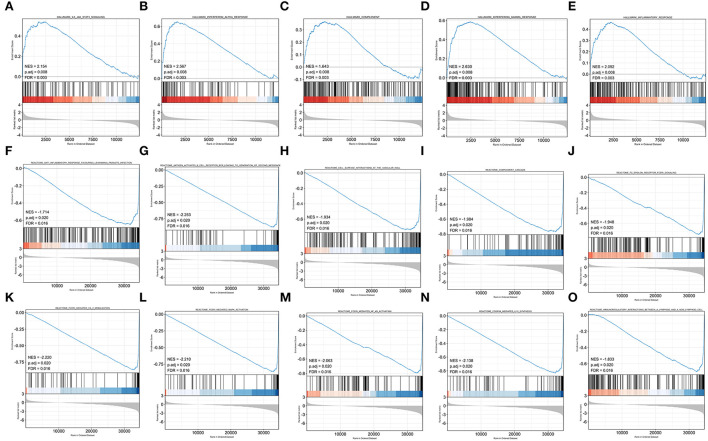
Gene set enrichment analysis (GSEA) of abatacept treatment and SARS-CoV-2 infection. **(A–E)** Five representative gene sets enriched in whole blood with abatacept treatment. **(F–O)** Ten representative gene sets enriched in PBMC with SARS-CoV-2 infection.

To obtain insight into the effect of abatacept treatment and SARS-CoV-2 infection, GSEA analysis indicated several biological processes. The top 20 significant gene sets are shown in Tables 2, 3. We showed representative 5 gene sets mainly participated in immunity and inflammation pathways enriched in whole blood with abatacept treatment, such as interleukin (IL)-6 JAK STAT3 signaling, interferon-alpha response, and complement (Figures 2A–E), and representative 10 gene sets in PBMC with SARS-CoV-2 infection such as anti-inflammatory response favoring leishmania parasite infection, antigen-activated B cell receptor BCR leading to the generation of the second messenger, and complement cascade (Figures 2F–O).

**Table 2:** The top 20 gene sets of before abatacept treatment vs. post abatacept treatment in gene set enrichment analysis.

**Gene set follow link to MsigDB**	**NES**	**p.adjust**
HALLMARK_EPITHELIAL_MESENCHYMAL_TRANSITION	−2.2503268	0.00800256
HALLMARK_ESTROGEN_RESPONSE_EARLY	−2.2343682	0.00800256
HALLMARK_MYOGENESIS	−1.9364	0.00800256
HALLMARK_UV_RESPONSE_DN	−2.0701299	0.00800256
HALLMARK_ANGIOGENESIS	−1.7538897	0.00800256
HALLMARK_MYC_TARGETS_V2	2.43048893	0.00800256
HALLMARK_ESTROGEN_RESPONSE_LATE	−1.6053365	0.00800256
HALLMARK_IL6_JAK_STAT3_SIGNALING	2.15353377	0.00800256
HALLMARK_UNFOLDED_PROTEIN_RESPONSE	1.76621231	0.00800256
HALLMARK_INTERFERON_ALPHA_RESPONSE	2.56668673	0.00800256
HALLMARK_SPERMATOGENESIS	2.04311146	0.00800256
HALLMARK_ALLOGRAFT_REJECTION	2.51118144	0.00800256
HALLMARK_MITOTIC_SPINDLE	2.25991007	0.00800256
HALLMARK_COMPLEMENT	1.64338085	0.00800256
HALLMARK_E2F_TARGETS	3.47332809	0.00800256
HALLMARK_G2M_CHECKPOINT	3.38236362	0.00800256
HALLMARK_INFLAMMATORY_RESPONSE	2.0919423	0.00800256
HALLMARK_GLYCOLYSIS	1.66154511	0.00800256
HALLMARK_INTERFERON_GAMMA_RESPONSE	2.62992434	0.00800256
HALLMARK_MTORC1_SIGNALING	2.59070521	0.00800256

**Table 3:** The top 20 gene sets of COVID-19 patients vs. normal controls in gene set enrichment analysis.

**Gene set follow link to MSigDB**	**NES**	**Padj**
REACTOME_ACTIVATION_OF_ANTERIOR_HOX_GENES_IN_HINDBRAIN_DEVELOPMENT_DURING_EARLY_EMBRYOGENESIS	−1.3797008	0.02003569
REACTOME_AMYLOID_FIBER_FORMATION	−1.4081053	0.02003569
REACTOME_ANTI_INFLAMMATORY_RESPONSE_FAVOURING_LEISHMANIA_PARASITE_INFECTION	−1.7136278	0.02003569
REACTOME_ANTIGEN_ACTIVATES_B_CELL_RECEPTOR_BCR_LEADING_TO_GENERATION_OF_SECOND_MESSENGERS	−2.2527823	0.02003569
REACTOME_ANTIMICROBIAL_PEPTIDES	−1.4196679	0.02003569
REACTOME_B_WICH_COMPLEX_POSITIVELY_REGULATES_RRNA_EXPRESSION	−1.413416	0.02003569
REACTOME_BASE_EXCISION_REPAIR	−1.4071511	0.02003569
REACTOME_BINDING_AND_UPTAKE_OF_LIGANDS_BY_SCAVENGER_RECEPTORS	−2.1092033	0.02003569
REACTOME_CELL_SURFACE_INTERACTIONS_AT_THE_VASCULAR_WALL	−1.934295	0.02003569
REACTOME_COLLAGEN_FORMATION	−1.4346929	0.02003569
REACTOME_COMPLEMENT_CASCADE	−1.9839443	0.02003569
REACTOME_DEGRADATION_OF_THE_EXTRACELLULAR_MATRIX	−1.4721928	0.02003569
REACTOME_ECM_PROTEOGLYCANS	−1.4484679	0.02003569
REACTOME_EXTRACELLULAR_MATRIX_ORGANIZATION	−1.3760813	0.02003569
REACTOME_FACTORS_INVOLVED_IN_MEGAKARYOCYTE_DEVELOPMENT_AND_PLATELET_PRODUCTION	−1.3827846	0.02003569
REACTOME_FC_EPSILON_RECEPTOR_FCERI_SIGNALING	−1.9478931	0.02003569
REACTOME_FCERI_MEDIATED_CA_2_MOBILIZATION	−2.2197513	0.02003569
REACTOME_FCERI_MEDIATED_MAPK_ACTIVATION	−2.2101849	0.02003569
REACTOME_FCERI_MEDIATED_NF_KB_ACTIVATION	−2.0625369	0.02003569
REACTOME_FCGAMMA_RECEPTOR_FCGR_DEPENDENT_PHAGOCYTOSIS	−2.0675858	0.02003569
REACTOME_FCGR3A_MEDIATED_IL10_SYNTHESIS	−2.1380411	0.02003569

### GO analysis of 59 DEGs

**Figure 3:**
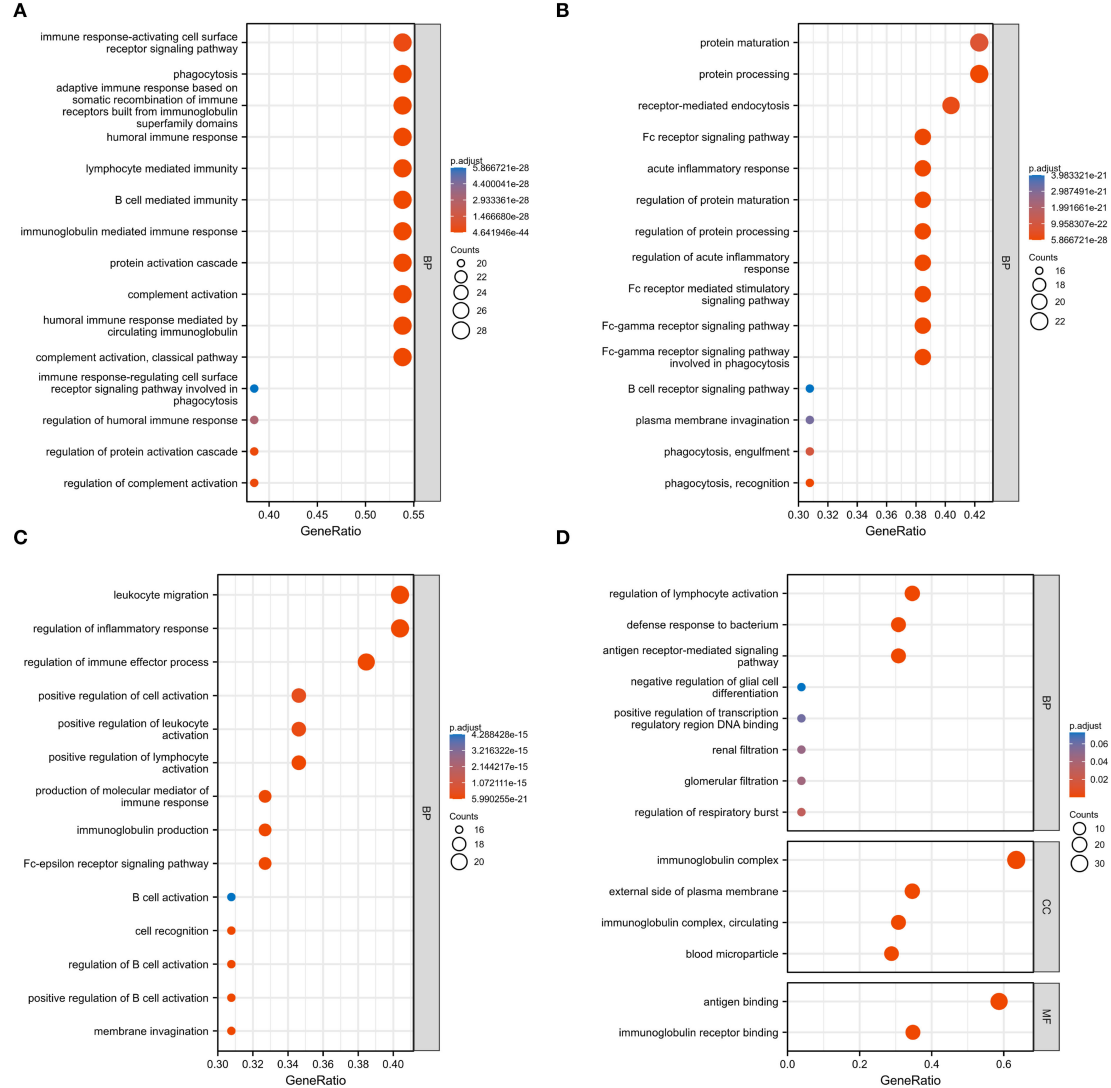
Gene ontology (GO) analysis of 59 common DEGs. **(A–D)** Fifty-eight GO terms were enriched. BP, biological process; CC, cellular component; MF, molecular function.

**Figure 4:**
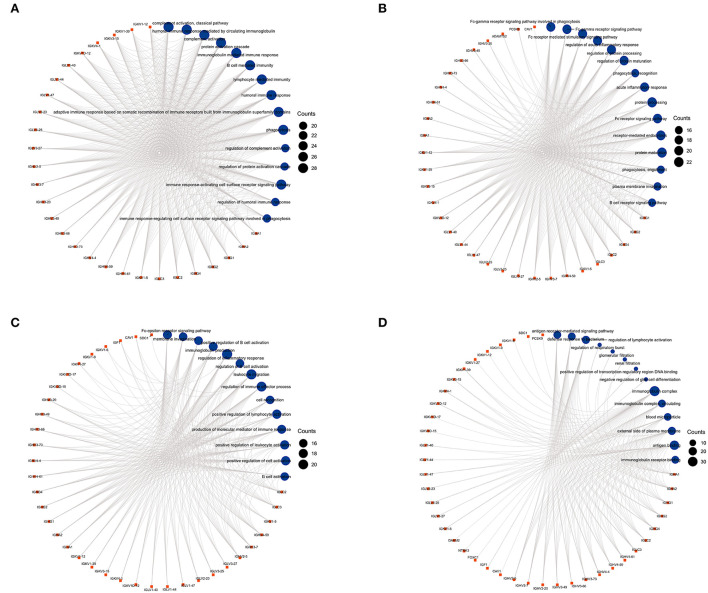
The circular diagrams of GO analysis of 59 common DEGs. **(A–D)** Fifty-eight GO terms were enriched. The arcs showed the corresponding relationship between DEGs (in red) and GO terms (in blue) in the GO analysis.

We used GO analysis to identify 58 GO terms of 59 DEGs upregulated in COVID-19 patients and downregulated post abatacept treatment, which included biological process (BP), cellular component (CC), and molecular function (MF) (Figure 3). Many immune-mediated pathways were enriched, such as the immune response-activating cell surface receptor signaling pathway, B cell-mediated immunity, lymphocyte-mediated immunity, and humoral immune response. The circular diagrams displayed the corresponding relationship between DEGs and GO terms (Figure 4). The predominantly related pathways of immune response mainly include the classical pathway of complement activation, immunoglobulin-mediated immune response, Fc-gamma receptor signaling pathway, immune response-activating signal transduction, and cell surface receptor signaling pathway. The Cluego tool of Cytoscape software was used to enrich immune-related pathways of 59 DEGs, such as the classical pathway of complement activation, humoral immune response mediated by circulating immunoglobulin and complement activation (Table 4).

**Table 4:** Gene ontology (immune system process) analysis of 59 common differentially expressed genes (top 15).

**Term**	**Description**	**Padj**
GO:0006958	Complement activation, classical pathway	1.1E-33
GO:0002455	Humoral immune response mediated by circulating immunoglobulin	7.3E-33
GO:0006956	Complement activation	5.7E-31
GO:0016064	Immunoglobulin mediated immune response	4.6E-28
GO:0019724	B cell mediated immunity	5.5E-28
GO:0030449	Regulation of complement activation	1.3E-22
GO:0002449	Lymphocyte mediated immunity	1.1E-21
GO:0002460	Adaptive immune response based on somatic recombination of immune receptors built from immunoglobulin superfamily domains	1.8E-21
GO:0002433	Immune response-regulating cell surface receptor signaling pathway involved in phagocytosis	5.9E-21
GO:0038096	Fc-gamma receptor signaling pathway involved in phagocytosis	5.9E-21
GO:0002920	Regulation of humoral immune response	6.2E-21
GO:0038094	Fc-gamma receptor signaling pathway	6.6E-21
GO:0002431	Fc receptor mediated stimulatory signaling pathway	1.1E-20
GO:0002757	Immune response-activating signal transduction	1.5E-18
GO:0002429	Immune response-activating cell surface receptor signaling pathway	1.5E-18

### PPI network construction and hub gene identification

**Figure 5:**
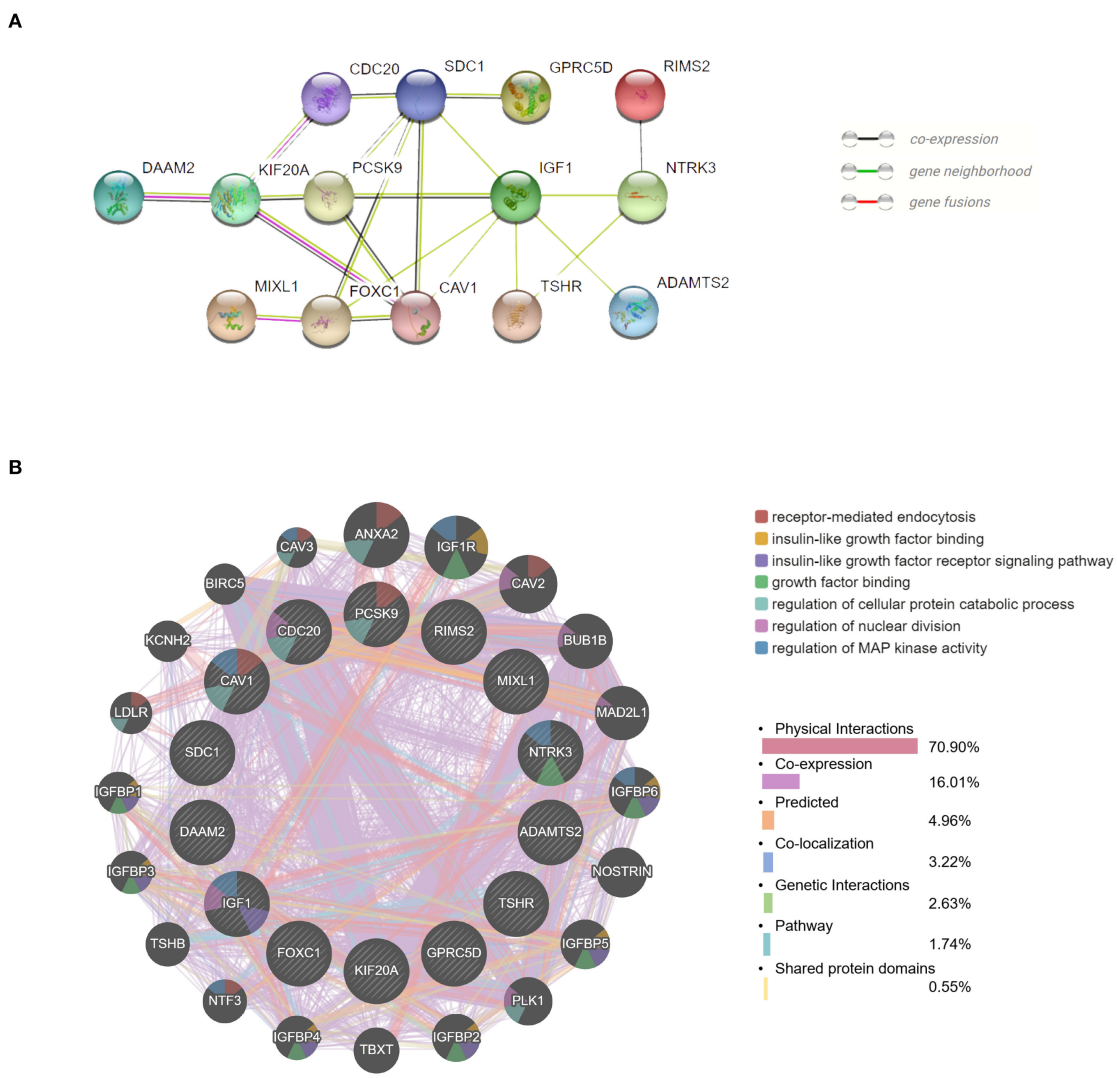
Protein–protein interaction (PPI) network and function analyses of hub genes. **(A)** The PPI network of the top 14 hub genes created by STRING. **(B)** The PPI network and function analyses of 14 hub genes based on the GeneMANIA database. The inner circle presents the hub genes and the outer circle shows the potential genes interacting with hub genes. The circle size indicates the correlation with the input hub genes.

We obtained the top 14 hub genes with the highest interaction degrees and constructed a PPI network using the STRING database (Figure 5A), which included DAAM2, KIF20A, PCSK9, MIXL1, CDC20, FOXC1, SDC1, CAV1, GPRC5D, IGF1, TSHR, RIMS2, NTRK3, and ADAMTS2. PPI network and function analyses were further analyzed for 14 hub genes. The results illustrate that the complex PPI network was with physical interactions of 70.90%, co-expression of 16.01%, co-localization of 3.22%, genetic interactions of 2.63%, predicted of 4.96%, shared protein domains of 0.55%, and a pathway of 1.74%. Receptor-mediated endocytosis, insulin-like growth factor binding, insulin-like growth factor receptor signaling pathway, growth factor binding, regulation of cellular protein catabolic process, regulation of nuclear division, and regulation of MAP kinase activity were identified as the main functions of those hub genes (Figure 5B).

### Validation of hub gene expression

**Figure 6:**
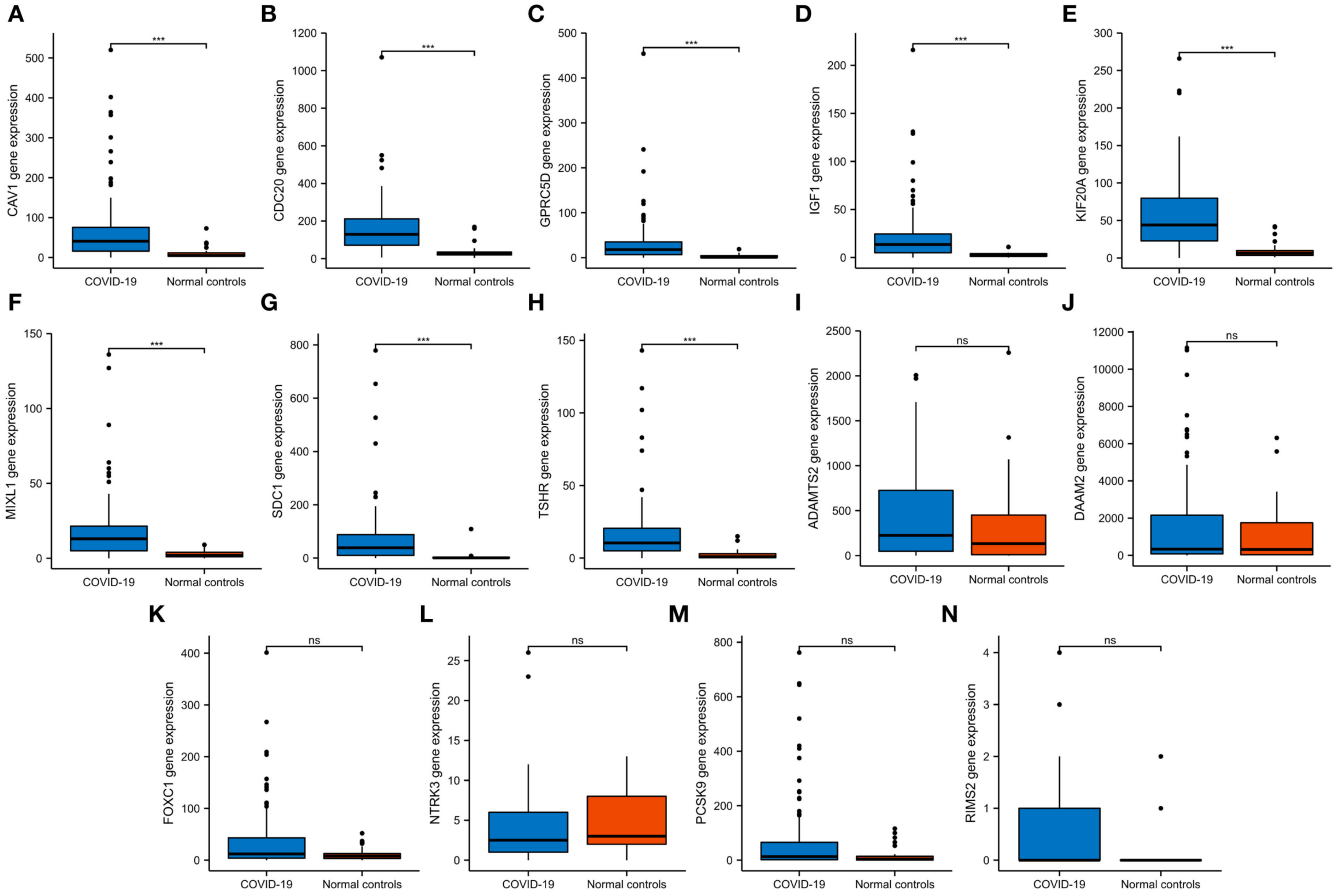
The expression validation of 14 hub genes. **(A–H)** In the GSE157103 dataset, the expression of CAV1, CDC20, GPRC5D, IGF1, KIF20A, MIXL1, SDC1, and TSHR genes was significantly higher in COVID-19 whole blood leukocytes than in normal controls, which was consistent with the GSE152418 dataset. **(I–N)** In the GSE157103 dataset, the expression of ADAMTS2, DAAM2, FOXC1, NTRK3, PCSK9, and RIMS2 genes has no significant differences in whole blood leukocyte samples between COVID-19 patients and normal controls. NS, no significance; ***P < 0.001.

We then verified the expression of 14 hub genes in the GSE157103 dataset. The expression of eight genes, including CAV1, CDC20, GPRC5D, IGF1, KIF20A, MIXL1, SDC1, and TSHR, was significantly upregulated in whole blood leukocyte samples of COVID-19 patients than normal controls. All of them were consistent with our previous analysis in the GSE152418 dataset (Figures 6A–H). The characteristics of the eight genes are described in Table 5. The expression of ADAMTS2, DAAM2, FOXC1, NTRK3, PCSK9, and RIMS2 genes has no significant difference between COVID-19 patients and normal controls (Figures 6I–N).

**Table 5:** Eight hub genes validated by GSE157103 dataset.

**Gene name/ID**	**Description**
CAV1/857	Caveolin 1
CDC20/991	Cell division cycle 20
GPRC5D/55507	G protein-coupled receptor class C group 5 member D
IGF1/3479	Insulin like growth factor 1
KIF20A/10112	Kinesin family member 20A
MIXL1/83881	Mix paired-like homeobox
SDC1/6382	Syndecan 1
TSHR/7253	Thyroid stimulating hormone receptor

### Construction of mRNA-miRNA network and immune correlation analysis

**Figure 7:**
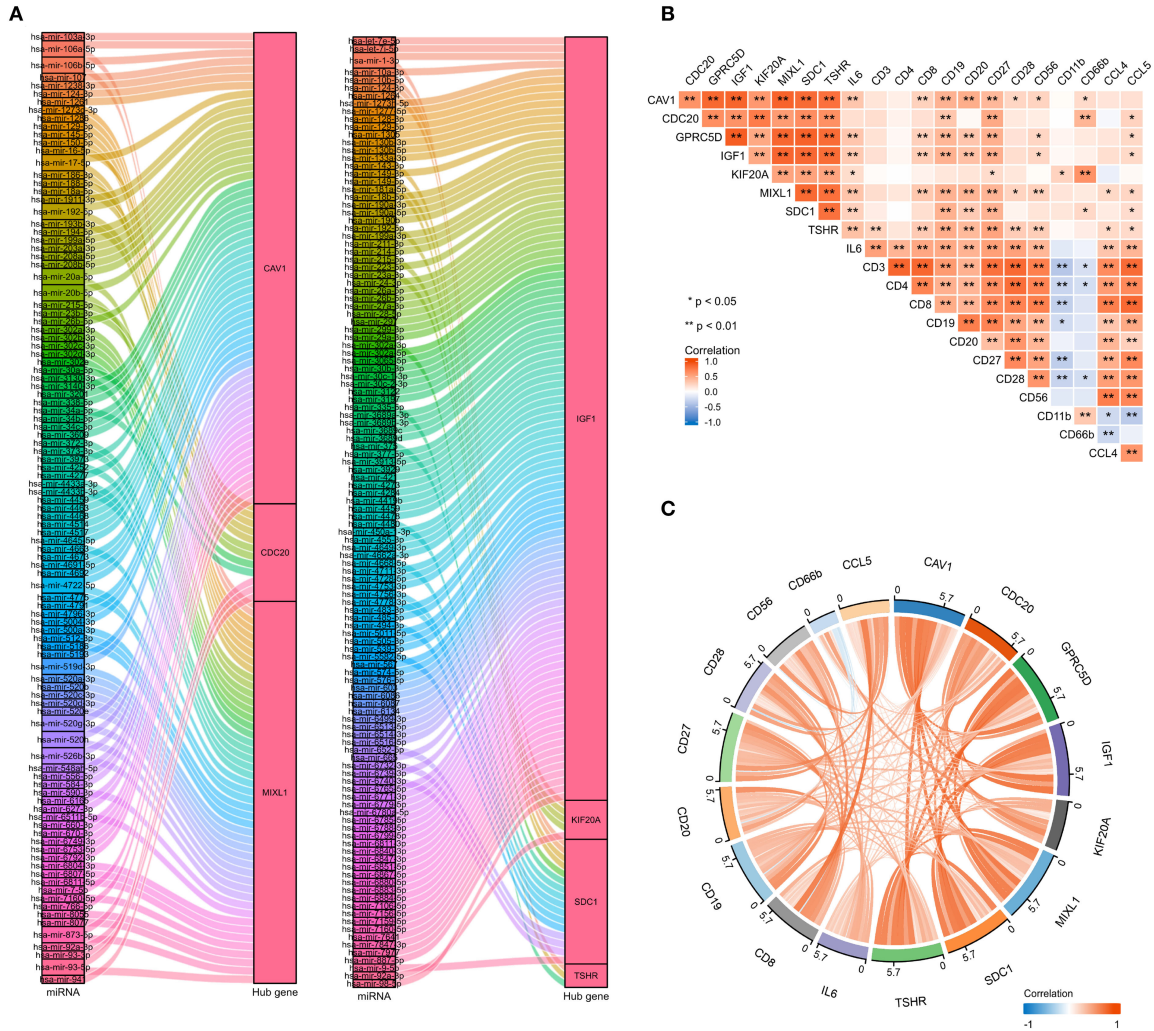
The mRNA-miRNA co-expressed interaction network constructed by Cytoscape and the correlation between hub genes and immunocyte-related molecular markers. **(A)** The mRNA-miRNA co-expressed networks including 230 target miRNAs for 7 hub genes, the prediction information for the GPRC5D gene was lacking in the NetworkAnalyst database. **(B)** The expression correlation among hub genes (CAV1, CDC20, GPRC5D, IGF1, KIF20A, MIXL1, SDC1, and TSHR), IL-6, and immunocyte-related genes (CD3, CD4, CD8, CD19, CD20, CD27, CD28, CD56, CD11b, CD66b, CCL4, and CCL5). Correspondence between biomarkers and immunocyte types: CD3: T cells; CD4: helper T cells; CD8: cytotoxic T cells; CD19, CD20, and CD27: B cells; CD28: activated T cells; CD56: natural killer cells; CD11b and CD66b: activated neutrophils. **(C)** The expression correlation among hub genes, IL-6, CD3, CD8, CD19, CD20, CD27, CD28, CD56, CD66b, and CCL5, had high correlations with hub genes. The red color indicates a positive correlation, and the blue color presents a negative correlation. *P < 0.05, **P < 0.01.

To further explore the potential mechanisms and regulating axis of hub genes in the process of abatacept-treated COVID-19, we established mRNA-miRNA co-expressed interaction networks using the NetworkAnalyst database and analyzed the correlation between hub genes and immunocyte-related molecular markers. In mRNA-miRNA co-expressed networks, 230 target miRNAs of seven hub genes without GPRC5D were included (Figure 7A). The expression of 8 hub genes was related to inflammatory factors and lymphocyte-related gene markers, including CD3, CD4, CD8, CD19, CD20, CD27, CD28, CD56, CD11b, CD66b, IL-6, CCL4, and CCL5. The correlations indicated the importance of immune cells participating in the immune-inflammation response (Figure 7B). The noticeable correlation between 8 hub genes and immune cell-related genes, including IL-6, CD3, CD8, CD19, CD20, CD27, CD28, CD56, CD66b, and CCL5, is also displayed in the annular chart (Figure 7C).

### Identification of drug candidates

To identify potential drug candidates for the treatment of COVID-19, we predicted the top 15 drug candidates targeting 8 hub genes such as deferoxamine, monobenzone, bicalutamide, trifluridine, and raloxifene (Supplementary Table 1).

### Tissue-specific and cell-specific expression of eight hub genes

The expression of 8 hub genes in multiple tissues was analyzed at RNA and protein levels. Remarkably, CDC20 RNA expression was highest in bone marrow, KIF20A was highest in the thymus, and MIXL1 was highest in the tonsil (Supplementary Figure 1). At the protein level, CAV1 was highly expressed in the lung, KIF20A was highly expressed in lymph nodes and bone marrow, GPRC5D was highly expressed in the spleen, and SDC1 was highly expressed in the tonsil (Supplementary Figure 2). CAV1 was highly expressed in lung tissue both at RNA and protein levels. The single-cell expression of 8 hub genes in lung tissues showed that CAV1, KIF20A, and SDC1 genes were highly expressed in alveolar cells. The CDC20, IGF1, and TSHR genes were highly expressed in endothelial cells (Supplementary Figure 2). The expression of 8 hub genes in immune cell subtypes was mainly in basophil, regulatory T cells, B cells, and NK cells (Supplementary Figure 3).

## Discussion

The SARS-CoV-2 is responsible for the COVID-19 pandemic, which has rapidly spread throughout the world and become a major threat to human beings (23). COVID-19 continues to expand in the pandemic form and is responsible for significant morbidity and mortality. The initial symptoms of COVID-19 are mainly fever, cough, myalgia, fatigue, and dyspnea. In addition, acute respiratory distress syndrome or multiple organ failure may gradually occur in terminal COVID-19 patients (24). COVID-19 patients requiring hospitalization were generally aged with multiple comorbidities. There is a relatively high rate of severe mortality in the early days of the epidemic, at 49 and 33%, respectively (25). Approximately, 14% of patients were severe cases that required ventilation in an intensive care unit (ICU), 5% were critical, and around 2.3% died in a report of 72,314 cases in China (26). While the treatment for severe patients is mainly symptomatic supportive therapy. Drug repositions are necessary to be accelerated for severe patients.

Angiotensin-converting enzyme-2 (ACE2) is a receptor for the binding and entry of the SARS-CoV-2 virus into cells (27). On binding to epithelial cells in the respiratory tract, SARS-CoV-2 starts replicating and migrating down to the airways and finally enters alveolar epithelial cells in the lung tissues. The rapid replication of SARS-CoV-2 in the lungs may trigger strong immune and inflammatory responses and produce large amounts of inflammatory cytokines, which leads to the cytokine storm, a major cause of patient death (28, 29). These uncontrolled inflammatory responses may lead to local and systemic tissue damage (30). Elevated pro-inflammatory cytokine is a typical profile in patients with COVID-19, such as IL-6, IL-1, and tumor necrosis factor (TNF)-α (31). Tocilizumab, as an anti-IL-6 receptor antibody, has performed its superiority in preventing severe outcomes such as mechanical ventilation and reducing death risk (32). Anakinra, Canakinumab, and Rilonacept are IL-1 blockades, which are currently used not only for therapy of RA and many other autoimmune rheumatic diseases but also for the treatment of the cytokine storm (33). TNF-α is an important mediator of other cytokines and chemokine production, so anti-TNFα therapy could be useful in COVID-19 (34). Similarly, these antirheumatic drugs might play a protective role in the development of the exaggerated immune-mediated inflammatory response associated with SARS-CoV-2 infection. The T-cell immune response is essential for protecting from COVID-19 and participates in abating innate immune responses involved in cytokine syndrome (31). In terms of abatacept, it can compete with CD28 for CD80/CD86 receptors, inhibiting its downstream inflammation reaction and suppressing the expression of other costimulatory molecules on antigen-presenting cells, resulting in a decrease in immune responses (35). Hence, viral inhibition is expected to be the most effective in the early disease course, while immunosuppressive treatment may be useful in the later stages to prevent severe disease. Multiple biological agents have shown their huge potential in the COVID-19 treatment (12).

We used bioinformatics to deeply analyze the RNA-Seq datasets GSE151161 and GSE152418, which included the samples of abatacept-treated RA and SARS-CoV-2-infected patients. First, we screened 129 DEGs in the GSE151161 dataset and 4,182 DEGs in the GSE152418 dataset. Venn diagram results identified 59 DEGs, upregulating in COVID-19 patients and downregulating in RA patients post abatacept treatment for 12 weeks. We then performed a GSEA analysis of all detected genes. We found that DEGs played an indispensable role in immunity and inflammation pathways. GSEA revealed that the primary mechanism of SARS-CoV-2 infection and abatacept treatment was the immune and inflammatory response. Subsequently, GO analysis of 59 DEGs revealed specific mechanisms of abatacept treating SARS-CoV-2 infection. The results indicate that 59 DEGs are involved in several specific mechanisms of inflammatory response activation, such as the immune response-activating cell surface receptor signaling pathway of BP, immunoglobulin complex of CC, and immunoglobulin receptor binding of MF. The arcs of the circular diagrams showed the corresponding relationship between DEGs and GO terms. These results reveal analogical mechanisms through which SARS-CoV-2 infection and abatacept treatment change the immune process.

Previous studies have shown that COVID-19 induced a poor immune response, leading to virus-induced pathology, or a hyperactive immune response that leads to cytokine storms associated with uncontrolled inflammation, severe pulmonary tissue damage, and even death in severe COVID-19 patients (36). Similar to previous studies, our GSEA analysis showed immune-related signal pathways, which were consistent with the pathogenesis of COVID-19 and RA.

The top genes with the highest degree of interaction in the PPI network were considered hub genes, which may be critical for the treatment of COVID-19 patients. In our study, functional analysis of 14 hub genes showed their associations with receptor-mediated endocytosis, insulin-like growth factor binding, insulin-like growth factor receptor signaling pathway, growth factor binding, regulation of cellular protein catabolic processes, regulation of nuclear division, and regulation of MAP kinase activity, which were related to virus entry, cell growth, regulation of protein catabolism, and mitosis. We confirmed the expression of 8 hub genes in the GSE152418 dataset, including CAV1, CDC20, GPRC5D, IGF1, KIF20A, MIXL1, SDC1, and TSHR. A previous study found that a high level of SDC1 and a low level of IGF1 increased mortality, which are potential biomarkers of severe COVID-19 (37, 38). In addition, CAV1, CDC20, and KIF20A were also identified as hub genes in COVID-19 by other researchers (39–41). Our study confirmed and complemented previous studies to some extent.

We identified 15 drug molecules targeted at 8 hub genes. Consistent with our results, deferoxamine, bicalutamide, trifluridine, raloxifene, etoposide, methotrexate, and progesterone were considered potential drugs for COVID-19 in previous studies among the top 15 drug candidates (42–48). However, the therapeutic effect of these candidate drugs on COVID-19 needs further study.

It is generally accepted that increased pro-inflammatory cytokines are associated with disease severity. Blocking immunity-induced inflammation was essential for reversing immunopathology. Our mRNA-miRNA co-expressed interaction networks revealed the potential regulatory mechanism of targeting genes, which guides the follow-up study of COVID-19 treatment mechanisms. A cytokine storm is potentially fatal and is characterized by the high-level activation of immune cells and the excessive production of massive inflammatory cytokines. A comparison between ICU and non-ICU patients showed that plasma concentrations of IL2, IL7, IL10, and TNFα were higher in ICU patients than in non-ICU patients (49). A retrospective study suggested elevated IL-6 was possibly fatality contributors and that mortality might be due to viral-driven hyperinflammation (50). Cytotoxic T cells, B cells, NK cells, and neutrophils can trigger SARS-CoV-2 infection-mediated CRS (51). Increased neutrophil number and neutrophil/lymphocyte ratio are usually accompanied by advanced severity and poor clinical outcomes (52). In some COVID-19 patients, the feature of severe SARS-CoV-2 infection is lymphopenia with severely exhausted CD4+ T cells, CD8+ T cells, B cells, and NK cell counts (51). Some studies also analyzed histological changes induced by SARS-CoV-2 infection. Tissue samples obtained from COVID-19 patients showed marked infiltration of various T-cell subclasses in the lungs (53). Thus, a dysregulated and excessive immune response not only initiates immune infiltration but also results in extensive inflammation and immunopathology through the induction of proinflammatory cytokines (51). Our study found a high correlation between 8 target genes and immune cell markers (CD8, CD19, CD20, CD27, CD56, and CD66b), which further demonstrated the crucial role of 8 target genes in COVID-19 treatment.

Overall, we provided insights into the SARS-CoV-2 infection and the immunomodulatory mechanisms of abatacept treatment. Our study suggested that abatacept was a potential strategy for severe COVID-19 based on the intersection of DEGs, the resemblance of antirheumatoid mechanisms and immune-inflammatory responses in COVID-19. Abatacept treatment could be a critical way to avoid an inflammatory storm in COVID-19 (13). Compared with the study of Julia et al., we conducted an analysis using larger sample size, identified potential biomarkers, and explored deeper mechanisms through which abatacept treats CODVID-19. More clinical trials of drug candidates should be tried to exert efficacy and tolerable safety in clinical therapy. The current study has several limitations. Only bioinformatics analysis was conducted in this study. We have further planned randomized and controlled trials (RCT) to support our conclusions.

## Data availability statement

Publicly available datasets were analyzed in this study. This data can be found at: GEO database, under accessions GSE151161, GSE152418, GSE157103.

## Author contributions

DY, HL, and QJ: conception and design. QJ: administrative support. DY, YC, and QJ: provision of study materials. HL, YC, and WR: collection and assembly of data. DY, WR, MD, and CL: data analysis and interpretation. DY and HL: manuscript writing. All authors approved the final manuscript.

## Funding

This study was supported by Natural Science Foundation of Shanxi Province: 20210302123287.

## Conflict of interest

The authors declare that the research was conducted in the absence of any commercial or financial relationships that could be construed as a potential conflict of interest.

## Publisher's note

All claims expressed in this article are solely those of the authors and do not necessarily represent those of their affiliated organizations, or those of the publisher, the editors and the reviewers. Any product that may be evaluated in this article, or claim that may be made by its manufacturer, is not guaranteed or endorsed by the publisher.

## References

[1] Huang X Wei F Hu L Wen L Chen K. Epidemiology and clinical characteristics of COVID-19. Arch Iran Med. (2020) 23:268–71. 10.34172/aim.2020.09 32271601

[2] Stasi C Fallani S Voller F Silvestri C. Treatment for COVID-19: an overview. Eur J Pharmacol. (2020) 889:173644. 10.1016/j.ejphar.2020.173644 33053381 PMC7548059

[3] Toor SM Saleh R Sasidharan Nair V Taha RZ Elkord E. T-cell responses and therapies against SARS-CoV-2 infection. Immunology. (2021) 162:30–43. 10.1111/imm.13262 32935333 PMC7730020

[4] Diao B Wang C Tan Y Chen X Liu Y Ning L. Reduction and functional exhaustion of T cells in patients with coronavirus disease 2019 (COVID-19). Front Immunol. (2020) 11:827. 10.3389/fimmu.2020.00827 32425950 PMC7205903

[5] Vardhana SA Wolchok JD. The many faces of the anti-COVID immune response. J Exp Med. (2020) 217:e20200678. 10.1084/jem.20200678 32353870 PMC7191310

[6] Shi Y Wang Y Shao C Huang J Gan J Huang X. COVID-19 infection: the perspectives on immune responses. Cell Death Differ. (2020) 27:1451–4. 10.1038/s41418-020-0530-3 32205856 PMC7091918

[7] Zhang C Wu Z Li JW Zhao H Wang GQ. Cytokine release syndrome in severe COVID-19: interleukin-6 receptor antagonist tocilizumab may be the key to reduce mortality. Int J Antimicrob Agents. (2020) 55:105954. 10.1016/j.ijantimicag.2020.105954 32234467 PMC7118634

[8] Alkofide H Almohaizeie A Almuhaini S Alotaibi B Alkharfy KM. Tocilizumab and systemic corticosteroids in the management of patients with COVID-19: a systematic review and meta-analysis. Int J Infect Dis. (2021) 110:320–9. 10.1016/j.ijid.2021.07.021 34273515 PMC8278842

[9] Blair HA Deeks ED. Abatacept: a review in rheumatoid arthritis. Drugs. (2017) 77:1221–33. 10.1007/s40265-017-0775-4 28608166

[10] Pombo-Suarez M Gomez-Reino JJ. Abatacept for the treatment of rheumatoid arthritis. Expert Rev Clin Immunol. (2019) 15:319–26. 10.1080/1744666X.2019.1579642 30730220

[11] Tamura N Azuma T Misaki K Yamaguchi R Hirano F Sugiyama E. Effectiveness and safety of subcutaneous abatacept in biologic-naive RA patients at week 52: a Japanese multicentre investigational study (ORIGAMI study). Mod Rheumatol. (2021) 10.1093/mr/roab090 34915575

[12] Kawazoe M Kihara M Nanki T. Antirheumatic drugs against COVID-19 from the perspective of rheumatologists. Pharmaceuticals. (2021) 14:1256. 10.3390/ph14121256 34959657 PMC8705607

[13] Julia A Bonafonte-Pardas I Gomez A Lopez-Lasanta M Lopez-Corbeto M Martinez-Mateu SH. Targeting of the CD80/86 proinflammatory axis as a therapeutic strategy to prevent severe COVID-19. Sci Rep. (2021) 11:11462. 10.1038/s41598-021-90797-0 34075090 PMC8169841

[14] Love MI Huber W Anders S. Moderated estimation of fold change and dispersion for RNA-seq data with DESeq2. Genome Biol. (2014) 15:550. 10.1186/s13059-014-0550-8 25516281 PMC4302049

[15] Yu G Wang LG Han Y He QY. clusterProfiler: an R package for comparing biological themes among gene clusters. OMICS. (2012) 16:284–7. 10.1089/omi.2011.0118 22455463 PMC3339379

[16] Bindea G Mlecnik B Hackl H Charoentong P Tosolini M Kirilovsky A. ClueGO: a Cytoscape plug-in to decipher functionally grouped gene ontology and pathway annotation networks. Bioinformatics. (2009) 25:1091–3. 10.1093/bioinformatics/btp101 19237447 PMC2666812

[17] Shannon P Markiel A Ozier O Baliga NS Wang JT Ramage D. Cytoscape: a software environment for integrated models of biomolecular interaction networks. Genome Res. (2003) 13:2498–504. 10.1101/gr.1239303 14597658 PMC403769

[18] Szklarczyk D Gable AL Lyon D Junge A Wyder S Huerta-Cepas J. STRING v11: protein-protein association networks with increased coverage, supporting functional discovery in genome-wide experimental datasets. Nucleic acids research. (2019) 47:D607–13. 10.1093/nar/gky1131 30476243 PMC6323986

[19] Warde-Farley D Donaldson SL Comes O Zuberi K Badrawi R Chao P. The GeneMANIA prediction server: biological network integration for gene prioritization and predicting gene function. Nucleic Acids Res. (2010) 38:W214–20. 10.1093/nar/gkq537 20576703 PMC2896186

[20] Uhlen M Fagerberg L Hallstrom BM Lindskog C Oksvold P Mardinoglu A. Proteomics. Tissue-based map of the human proteome. Science. (2015) 347:1260419. 10.1126/science.1260419 25613900

[21] Zhou G Soufan O Ewald J Hancock REW Basu N Xia J. NetworkAnalyst 3.0: a visual analytics platform for comprehensive gene expression profiling and meta-analysis. Nucleic Acids Res. (2019) 47:W234–W41. 10.1093/nar/gkz240 30931480 PMC6602507

[22] Xie Z Bailey A Kuleshov MV Clarke DJB Evangelista JE Jenkins SL. Gene set knowledge discovery with enrichr. Curr Protoc. (2021) 1:e90. 10.1002/cpz1.90 33780170 PMC8152575

[23] Jin X Lian JS Hu JH Gao J Zheng L Zhang YM. Epidemiological, clinical and virological characteristics of 74 cases of coronavirus-infected disease 2019 (COVID-19) with gastrointestinal symptoms. Gut. (2020) 69:1002–9. 10.1136/gutjnl-2020-320926 32213556 PMC7133387

[24] Liu C Zhao Y Okwan-Duodu D Basho R Cui X. COVID-19 in cancer patients: risk, clinical features, and management. Cancer Biol Med. (2020) 17:519–27. 10.20892/j.issn.2095-3941.2020.0289 32944387 PMC7476081

[25] Buckner FS McCulloch DJ Atluri V Blain M McGuffin SA Nalla AK. Clinical features and outcomes of 105 hospitalized patients with COVID-19 in Seattle, Washington. Clin Infect Dis. (2020) 71:2167–73. 10.1093/cid/ciaa632 32444880 PMC7314181

[26] Wu Z McGoogan JM. Characteristics of and important lessons from the coronavirus disease 2019 (COVID-19) outbreak in China: summary of a report of 72314 cases from the Chinese center for disease control and prevention. JAMA. (2020) 323:1239–42. 10.1001/jama.2020.2648 32091533

[27] Shang J Wan Y Luo C Ye G Geng Q Auerbach A. Cell entry mechanisms of SARS-CoV-2. Proc Natl Acad Sci USA. (2020) 117:11727–34. 10.1073/pnas.2003138117 32376634 PMC7260975

[28] Chen G Wu D Guo W Cao Y Huang D Wang H. Clinical and immunological features of severe and moderate coronavirus disease 2019. J Clin Investig. (2020) 130:2620–9. 10.1172/JCI137244 32217835 PMC7190990

[29] Hu B Guo H Zhou P Shi ZL. Characteristics of SARS-CoV-2 and COVID-19. Nat Rev Microbiol. (2021) 19:141–54. 10.1038/s41579-020-00459-7 33024307 PMC7537588

[30] Anka AU Tahir MI Abubakar SD Alsabbagh M Zian Z Hamedifar H. Coronavirus disease 2019 (COVID-19): an overview of the immunopathology, serological diagnosis and management. Scand J Immunol. (2021) 93:e12998. 10.1111/sji.12998 33190302 PMC7744910

[31] Luo XH Zhu Y Mao J Du RC. T cell immunobiology and cytokine storm of COVID-19. Scand J Immunol. (2021) 93:e12989. 10.1111/sji.12989 33113222 PMC7645942

[32] Zhang C Jin H Wen YF Yin G. Efficacy of COVID-19 treatments: a bayesian network meta-analysis of randomized controlled trials. Front Public Health. (2021) 9:729559. 10.3389/fpubh.2021.729559 34650951 PMC8506153

[33] Soy M Keser G Atagunduz P Tabak F Atagunduz I Kayhan S. Cytokine storm in COVID-19: pathogenesis and overview of anti-inflammatory agents used in treatment. Clin Rheumatol. (2020) 39:2085–94. 10.1007/s10067-020-05190-5 32474885 PMC7260446

[34] Gonzalez-Gay MA Castaneda S Ancochea J. Biologic therapy in COVID-19. Arch Bronconeumol. (2021) 57:1–2. 10.1016/j.arbres.2020.06.007 34629622 PMC7318980

[35] Liu M Yu Y Hu S. A review on applications of abatacept in systemic rheumatic diseases. Int Immunopharmacol. (2021) 96:107612. 10.1016/j.intimp.2021.107612 33823429

[36] Chen N Zhou M Dong X Qu J Gong F Han Y. Epidemiological and clinical characteristics of 99 cases of 2019 novel coronavirus pneumonia in Wuhan, China: a descriptive study. Lancet. (2020) 395:507–13. 10.1016/S0140-6736(20)30211-7 32007143 PMC7135076

[37] Ilias I Diamantopoulos A Botoula E Athanasiou N Zacharis A Tsipilis S. Covid-19 and growth hormone/insulin-like growth factor 1: study in critically and non-critically ill patients. Front Endocrinol. (2021) 12:644055. 10.3389/fendo.2021.644055 34220703 PMC8242942

[38] Zhang D Li L Chen Y Ma J Yang Y Aodeng S. Syndecan-1, an indicator of endothelial glycocalyx degradation, predicts outcome of patients admitted to an ICU with COVID-19. Mol Med. (2021) 27:151. 10.1186/s10020-021-00412-1 34861818 PMC8640509

[39] Auwul MR Rahman MR Gov E Shahjaman M Moni MA. Bioinformatics and machine learning approach identifies potential drug targets and pathways in COVID-19. Brief Bioinform. (2021) 22:bbab120. 10.1093/bib/bbab120 33839760 PMC8083354

[40] Bharne D. A protein interactions map of multiple organ systems associated with COVID-19 disease. Genomics Inform. (2021) 19:e14. 10.5808/gi.20078 34261299 PMC8261268

[41] Chen JC Xie TA Lin ZZ Li YQ Xie YF Li ZW. Identification of key pathways and genes in SARS-CoV-2 infecting human intestines by bioinformatics analysis. Biochem Genet. (2021) 60:1076–94. 10.1007/s10528-021-10144-w 34787756 PMC8596852

[42] Hamizi K Aouidane S Belaaloui G. Etoposide-based therapy for severe forms of COVID-19. Med Hypotheses. (2020) 142:109826. 10.1016/j.mehy.2020.109826 32416415 PMC7207128

[43] Elzupir AO. Molecular docking and dynamics investigations for identifying potential inhibitors of the 3-chymotrypsin-like protease of SARS-CoV-2: repurposing of approved pyrimidonic pharmaceuticals for COVID-19 treatment. Molecules. (2021) 26:7458. 10.3390/molecules26247458 34946540 PMC8707611

[44] Goren A Wambier CG Herrera S McCoy J Vano-Galvan S Gioia F. Anti-androgens may protect against severe COVID-19 outcomes: results from a prospective cohort study of 77 hospitalized men. J Eur Acad Dermatol Venereol. (2021) 35:e13–e5. 10.1111/jdv.16953 32977363 PMC7536996

[45] Hong S Chang J Jeong K Lee W. Raloxifene as a treatment option for viral infections. J Microbiol. (2021) 59:124–31. 10.1007/s12275-021-0617-7 33527314 PMC7849956

[46] Mahil SK Bechman K Raharja A Domingo-Vila C Baudry D Brown MA. The effect of methotrexate and targeted immunosuppression on humoral and cellular immune responses to the COVID-19 vaccine BNT162b2: a cohort study. Lancet Rheumatol. (2021) 3:e627–e37. 10.1016/S2665-9913(21)00212-5 34258590 PMC8266273

[47] Shah SB. COVID-19 and progesterone: Part 2. Unraveling High Severity, Immunity Patterns, Immunity grading, Progesterone and its potential clinical use. Endocr Metab Sci. (2021) 5:100110. 10.1016/j.endmts.2021.100110 34396354 PMC8349364

[48] Poonkuzhi Naseef P Elayadeth-Meethal M Mohammed Salim KT Anjana A Muhas C Abdul Vajid K. Therapeutic potential of induced iron depletion using iron chelators in Covid-19. Saudi J Biol Sci. (2022) 29:1947–56. 10.1016/j.sjbs.2021.11.061 34924800 PMC8666385

[49] Huang C Wang Y Li X Ren L Zhao J Hu Y. Clinical features of patients infected with 2019 novel coronavirus in Wuhan, China. Lancet. (2020) 395:497–506. 10.1016/S0140-6736(20)30183-5 31986264 PMC7159299

[50] Mehta P McAuley DF Brown M Sanchez E Tattersall RS Manson JJ. COVID-19: consider cytokine storm syndromes and immunosuppression. Lancet. (2020) 395:1033–4. 10.1016/S0140-6736(20)30628-0 32192578 PMC7270045

[51] Tan Y Tang F. SARS-CoV-2-mediated immune system activation and potential application in immunotherapy. Med Res Rev. (2021) 41:1167–94. 10.1002/med.21756 33185926

[52] Tay MZ Poh CM Renia L MacAry PA Ng LFP. The trinity of COVID-19: immunity, inflammation and intervention. Nat Rev Immunol. (2020) 20:363–74. 10.1038/s41577-020-0311-8 32346093 PMC7187672

[53] Zinserling VA Semenova NY Markov AG Rybalchenko OV Wang J Rodionov RN. Inflammatory cell infiltration of adrenals in COVID-19. Hormone Metab Res. (2020) 52:639–41. 10.1055/a-1191-8094 32629518

