# Supplementary material for: Immunomodulatory mechanisms of abatacept: A therapeutic strategy for COVID-19
Source: Front Med (Lausanne). 2022 Jul 25;9:951115. doi: 10.3389/fmed.2022.951115 (PMC9357915; doi:10.3389/fmed.2022.951115)
Supplement: Supplementary file 1 [file Table_1.docx]

| Drug name | Adjusted P-value | Combined Score | Genes |
| --- | --- | --- | --- |
| deferoxamine MCF7 DOWN | 0.004421 | 3897.964 | CDC20; KIF20A |
| monobenzone MCF7 DOWN | 0.004421 | 3657.624 | CDC20; KIF20A |
| bicalutamide CTD 00002279 | 0.004421 | 735.5486 | CDC20; IGF1; KIF20A |
| trifluridine MCF7 DOWN | 0.006968 | 2043.045 | CDC20; KIF20A |
| raloxifene CTD 00007367 | 0.006968 | 265.043 | CAV1; SDC1; IGF1; KIF20A |
| 0173570-0000 PC3 DOWN | 0.007444 | 1457.249 | CDC20; KIF20A |
| etoposide MCF7 DOWN | 0.007444 | 1266.515 | CDC20; KIF20A |
| PNU-0293363 MCF7 DOWN | 0.007444 | 1202.252 | CDC20; KIF20A |
| methotrexate MCF7 DOWN | 0.007444 | 1143.566 | CDC20; KIF20A |
| benzo[a]pyrene CTD 00005488 | 0.007444 | 214.7407 | CDC20; CAV1; SDC1; IGF1; KIF20A; MIXL1; TSHR |
| Nitroprusside CTD 00006411 | 0.008545 | 973.222 | CAV1; IGF1 |
| ciclopirox HL60 DOWN | 0.008545 | 932.6101 | CDC20; KIF20A |
| ciclopirox MCF7 DOWN | 0.009105 | 827.0638 | CDC20; KIF20A |
| 5109870 MCF7 DOWN | 0.009105 | 811.5074 | CDC20; KIF20A |
| progesterone CTD 00006624 | 0.009449 | 125.5594 | CDC20; CAV1; SDC1; IGF1; KIF20A |

Supplement Table 1：Top 15 drug candidates combined with 8 hub genes.
