# Supplementary material for: Immunomodulatory mechanisms of abatacept: A therapeutic strategy for COVID-19
Source: Front Med (Lausanne). 2022 Jul 25;9:951115. doi: 10.3389/fmed.2022.951115 (PMC9357915; doi:10.3389/fmed.2022.951115)
Supplement: Supplementary file 3 [file Data_Sheet_2.DOCX]

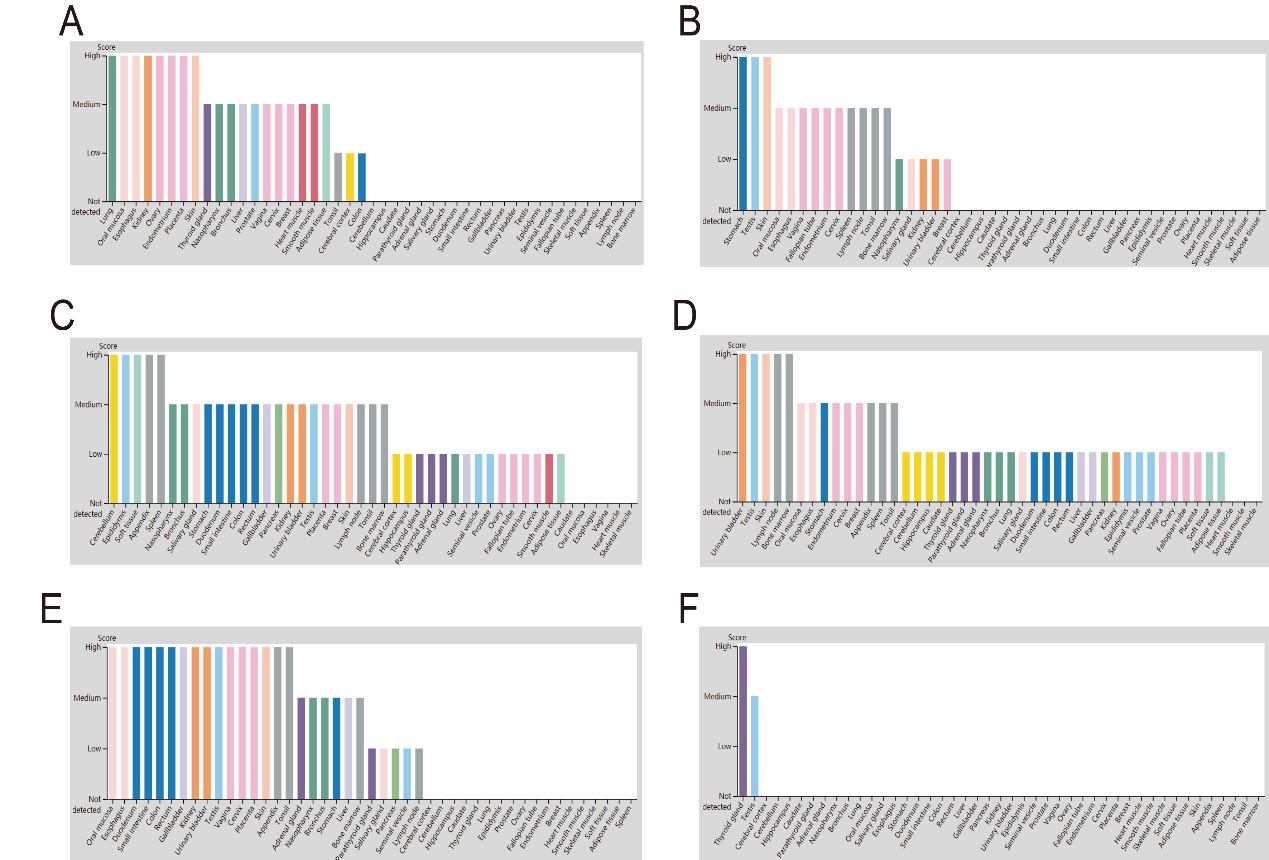


Sup Figure 2: The expression of 8 hub genes in multiple tissues. (A-F) The protein expression of CAV1, CDC20, GPRC5D, KIF20A, SDC1, and TSHR in HPA database.
