# Supplementary material for: Immunomodulatory mechanisms of abatacept: A therapeutic strategy for COVID-19
Source: Front Med (Lausanne). 2022 Jul 25;9:951115. doi: 10.3389/fmed.2022.951115 (PMC9357915; doi:10.3389/fmed.2022.951115)
Supplement: Supplementary file 4 [file Data_Sheet_3.docx]

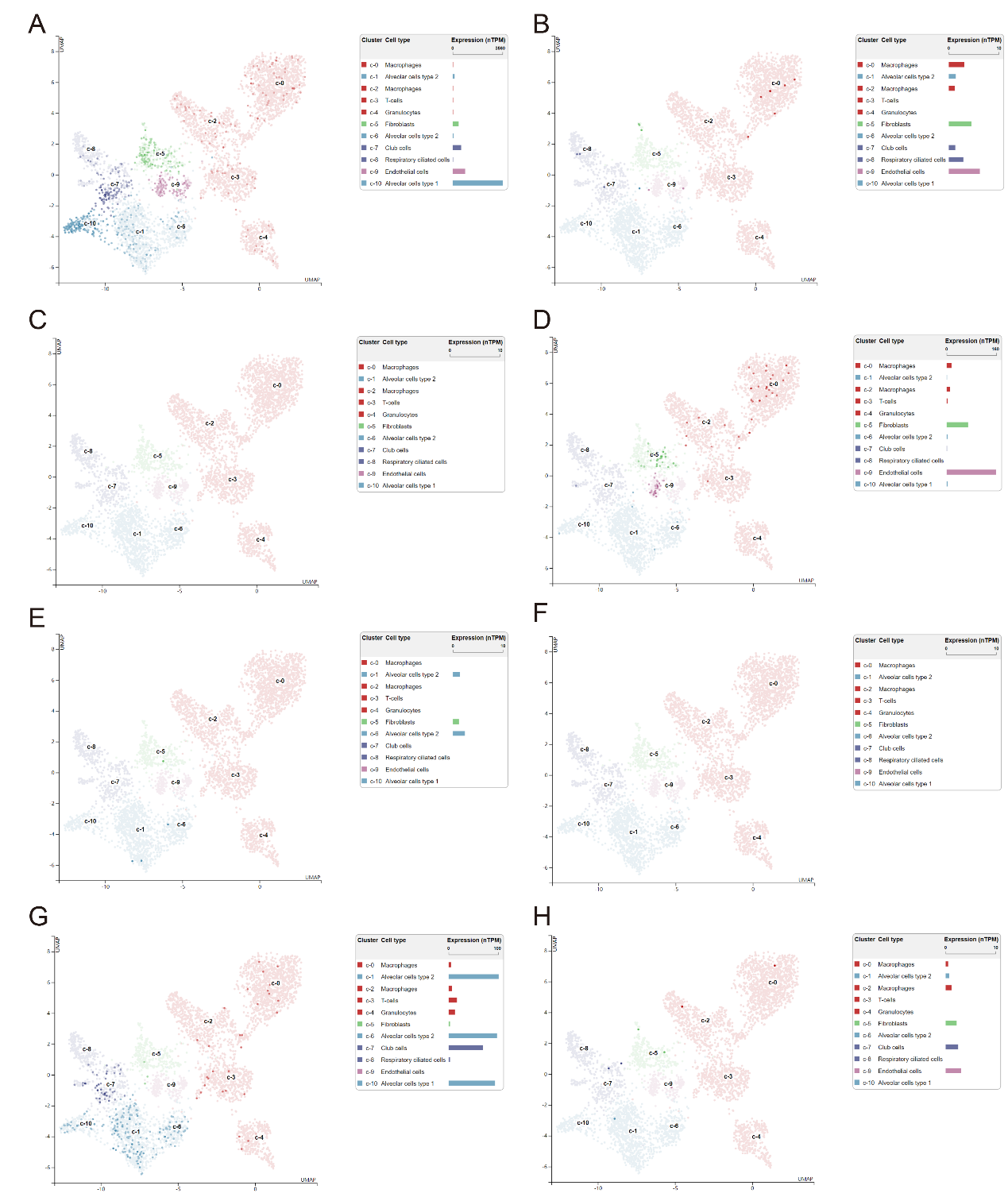


Sup Figure 3: The sing-cell expression of 8 hub genes in lung tissues. The RNA expression of CAV1 (A), CDC20 (B), GPRC5D (C), IGF1 (D), KIF20A (E), MIXL1 (F), SDC1 (G), and TSHR (H) in macrophage, alveolar cells type 2, T cells, granulocyte, fibroblast, club cell, respiratory ciliated cell, endothelial cell, and alveolar cells type 1 of lung tissue.
