# Supplementary material for: Immunomodulatory mechanisms of abatacept: A therapeutic strategy for COVID-19
Source: Front Med (Lausanne). 2022 Jul 25;9:951115. doi: 10.3389/fmed.2022.951115 (PMC9357915; doi:10.3389/fmed.2022.951115)
Supplement: Supplementary file 5 [file Data_Sheet_4.docx]

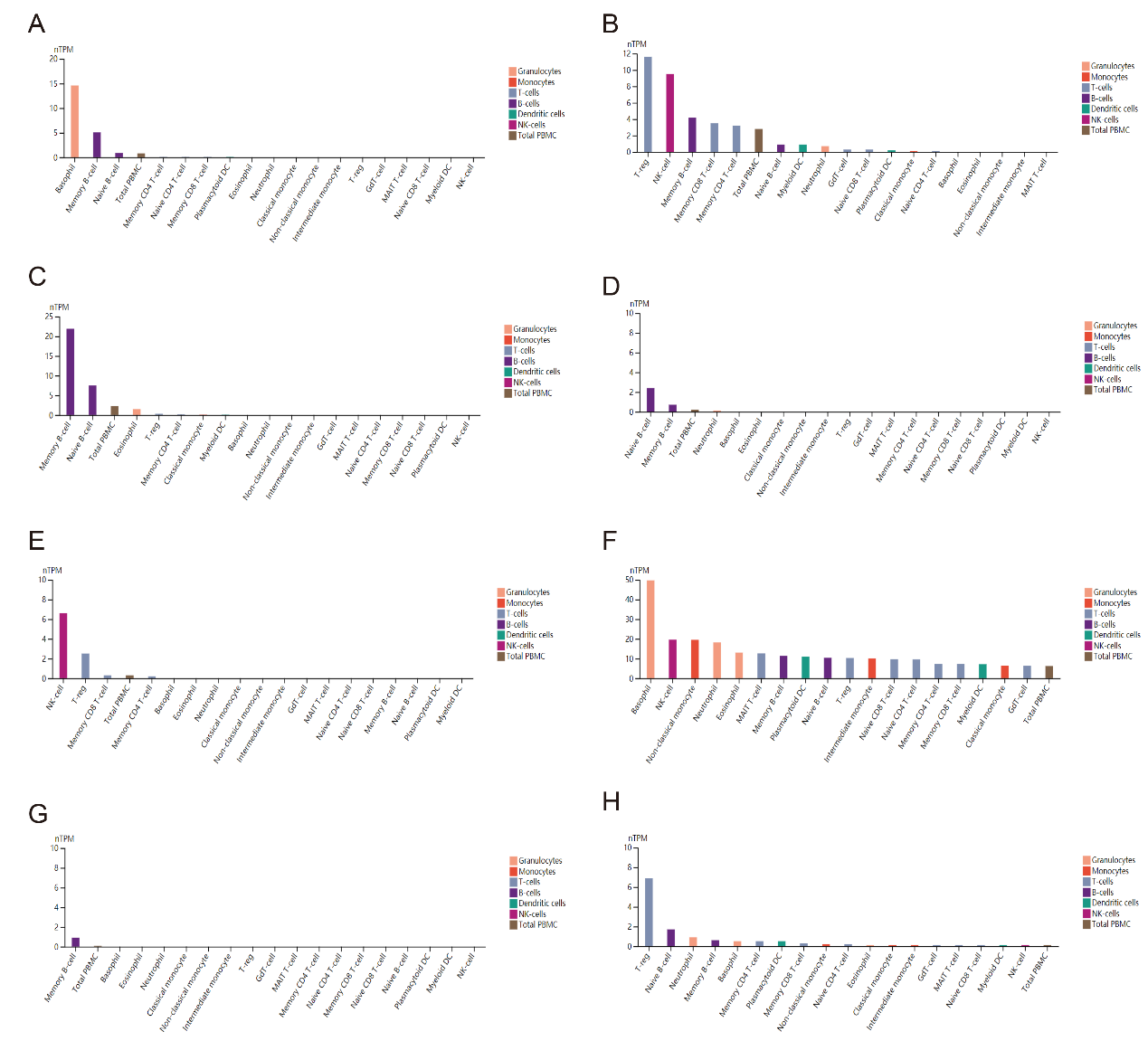


Sup Figure 4: The expression of 8 hub genes in immune cell subtypes. The RNA expression of CAV1 (A), CDC20 (B), GPRC5D (C), IGF1 (D), KIF20A (E), MIXL1 (F), SDC1 (G), and TSHR (H) in immune cell subtypes including DC, myeloid DC, memory CD8 T cell, natural killer (NK) cell, total PBMC, basophil, eosinophil, neutrophil, classical monocyte, non-classical monocyte, intermediate monocyte, regulatory T cell, gd T cell, MAIT cell, memory CD4 T cell, naïve CD4 T cell, naive CD8 T cell, memory B cell, and naive B cell.
